# Marker Assisted Transfer of Two Powdery Mildew Resistance Genes *PmTb7A*.*1* and *PmTb7A*.*2* from *Triticum boeoticum* (Boiss.) to *Triticum aestivum* (L.)

**DOI:** 10.1371/journal.pone.0128297

**Published:** 2015-06-11

**Authors:** Ahmed Fawzy Abdelnaby Elkot, Parveen Chhuneja, Satinder Kaur, Manny Saluja, Beat Keller, Kuldeep Singh

**Affiliations:** 1 School of Agricultural Biotechnology, Punjab Agricultural University, Ludhiana, 141 004, India; 2 Institute of Plant Biology, University of Zurich, Zurich, Switzerland; Institute for Sustainable Agriculture (IAS-CSIC), SPAIN

## Abstract

Powdery mildew (PM), caused by *Blumeria graminis* f. sp. *tritici*, is one of the important wheat diseases, worldwide. Two PM resistance genes, designated as *PmTb7A*.*1* and *PmTb7A*.*2*, were identified in *T*. *boeoticum* acc. pau5088 and mapped on chromosome 7AL approximately 48cM apart. Two resistance gene analogue (RGA)-STS markers *Ta7AL-4556232* and *7AL-4426363* were identified to be linked to the *PmTb7A*.*1* and *PmTb7A*.*2*, at a distance of 0.6cM and 6.0cM, respectively. In the present study, following marker assisted selection (MAS), the two genes were transferred to *T*. *aestivum* using *T*. *durum* as bridging species. As many as 12,317 florets of F_1_ of the cross *T*. *durum* /*T*. *boeoticum* were pollinated with *T*. *aestivum* lines PBW343-IL and PBW621 to produce 61 and 65 seeds, respectively, of three-way F_1_. The resulting F_1_s of the cross *T*. *durum/T*. *boeoticum//T*. *aestivum* were screened with marker flanking both the PM resistance genes *PmTb7A*.*1* and *PmTb7A*.*2* (foreground selection) and the selected plants were backcrossed to generate BC_1_F_1_. Marker assisted selection was carried both in BC_1_F_1_ and the BC_2_F_1_ generations. Introgression of alien chromatin in BC_2_F_1_ plants varied from 15.4 - 62.9 percent. Out of more than 110 BC_2_F_1_ plants showing introgression for markers linked to the two PM resistance genes, 40 agronomically desirable plants were selected for background selection for the carrier chromosome to identify the plants with minimum of the alien introgression. Cytological analysis showed that most plants have chromosome number ranging from 40-42. The BC_2_F_2_ plants homozygous for the two genes have been identified. These will be crossed to generate lines combining both the PM resistance genes but with minimal of the alien introgression. The PM resistance gene *PmTb7A*.*1* maps in a region very close to *Sr22*, a stem rust resistance gene effective against the race Ug99. Analysis of selected plants with markers linked to *Sr22* showed introgression of *Sr22* from *T*. *boeoticum* in several BC_2_F_1 _plants. Thus, in addition to PM resistance, these progeny might also carry resistance to stem rust race Ug99.

## Introduction

Bread wheat, *Triticum aestivum*, is the second most important staple food crop, providing ~20% of the calories and the protein requirements of the world population. The world average wheat yield is projected to rise from 3.2 tonnes/ha in the year 2013 to 3.4 tonnes/ha in 2025 but it must reach to 4.5 tonnes/ha to meet the global demand of 998 million tonnes or with the current growth rate of 0.9% per year an additional 46 million ha land needs to be added to meet the demand [1]. Among the several production constraints, diseases are the most important stress which can cause significant yield losses. In wheat, among the various foliar diseases, powdery mildew (PM) caused by the fungus *Blumeria graminis* f. sp. *tritici* is one of the most prevalent diseases worldwide. Damage caused by PM ranges from 13–34% when infection is low to moderate but under severe infection it could be more than 50% [2]—[5]. Severe epidemics of PM usually occur in areas with cool and humid climates [6]. The use of resistant cultivars is an efficient, economical and environmentally safe approach to control PM and reduce yield losses.

A number of PM resistance genes have been identified from cultivated wheat and its wild relatives. However, most of the resistance genes are race-specific and liable to resistance breaking down, once used in widely deployed cultivars. So far more than 78 PM resistance genes/alleles have been identified at 50 loci (*Pm1—Pm53*, *Pm18* = *Pm1c*, *Pm22* = *Pm1e*, *Pm23* = *Pm4c*, *Pm31* = *Pm21*) in wheat and its wild relatives [7]—[9]. Of the 50 loci, 11 have been mapped on the A genome, 26 on the B genome and 13 on the D genome of wheat. Twenty-seven of the PM genes/alleles have been transferred into wheat from wild species such as *T*. *monococcum* (three), *Ae*. *speltoides* (two), *Ae*. *tauschii* (four), *T*. *dicoccoides* (seven), *T*. *carthelicum* (two), *T*. *timopheevi* (three), *Secale cereale* (three), *Ae*. *ovata* (one), *Ae*. *umbellulata* (one), *Ae*. *longissima* (one), *Elytrigia intermedium* (one), *Haynaldia villosa* (one), and *Thinopyrum intermedium* (one) [8]—[19]. In addition to major genes, resistance to PM is also conferred by quantitative trait loci (QTL), and many of these have been mapped and confirmed as Meta QTL [20], [21]. Although a number of PM resistance genes and QTL have been identified and catalogued, the mildew pathogen continues to evolve new virulence as a result of mutation as well as genetic recombination due to sexual reproduction [22], [23]. Thus, the identification of new genes is essential for containing the disease.

In India, PM is prevalent in the northern and southern hill zones causing serious yield losses whereas in north western plains zone (NWPZ) of India, which constitutes the most productive wheat growing region, PM appears sporadically but causing significant yield losses [24]. Variability for PM resistance is limited in Indian germplasm [24], [25]. Most of the wheat varieties/germplasm lines recently developed, recently developed in India are susceptible to PM. Singh et al. [24] screened more than 400 germplasm lines over a period of four years at nine different locations across the country and only nine lines were reported resistant. This may be primarily because of increased use of ‘Veery’ derivatives. Such cultivars have *Pm8* gene which is susceptible to most of the PM races in India. Unlike rusts, wheat breeding programmes in NWPZ of India do not breed for PM resistance, primarily because of limitations of screening of the segregating populations against PM. Availability of resistance genes with closely linked DNA markers can help to integrate marker assisted selection of the desirable genes in wheat breeding programme.

*T*. *boeoticum*, (2*n* = 2*x* = 14, AA), a close relative of the A genome donor of wheat, harbours useful variability for many agronomically important traits including resistance to diseases [26]—[30]. Many of the PM resistance genes such as *Pm1a*, *Pm1b* and *Pm25* have been introgressed from diploid A genome progenitor species. The *Pm1* locus with five different alleles is located in chromosome 7AL [31], [32]. We identified *T*. *boeoticum* acc. pau5088 having resistance to PM and the resistance was conferred by two independent genes designated as *PmTb7A*.*1* and *PmTb7A*.*2*. Both the genes were mapped on chromosome 7AL at a distance of ~48cM [33]. Both genes were effective individually as well in combination against the PM races in Europe and India. These genes were mapped between the marker intervals wPt4553–*Xcfa2019* (4.3cM) and MAG1759–MAG2185b (1.4cM). Fine mapping of these genes showed that *PmTb7A*.*1* is a novel gene and *PmTb7A*.*2* could be a new allele of *Pm1* [34]. Using shotgun sequence assembly of chromosome 7A, RGA-STS markers *Ta7AL-4556232_rga* was identified to be linked with *PmTb7A*.*1* at a distance of 0.6cM and other RGA-STS markers *7AL-4426363_rga* and *7AL-4544237_rga* were identified to be linked to *PmTb7A*.*2* at distance of 6.0cM, though markers BE445506 and MAG1759 were closely linked at a distance of 0.9cM. The identification of molecular markers linked to resistance genes could facilitate marker-assisted selection and enable breeders to pyramid several major genes for PM resistance into a single cultivar.

Transfer of agronomically important genes even from closely related wild species is often associated with linkage drag, thus limiting commercialization of such genes. Stem rust resistance gene *Sr22* transferred from *T*. *boeoticum* confers resistance to *Puccinia graminis* f. sp. *tritici* race TTKSK (also known as Ug99) but could be deployed in a limited number of cultivars due to poor agronomic performance of lines carrying the resistance gene [35], though lines with shortened introgressed segment have now been generated in hexaploid wheat background and markers closely linked to *Sr22* identified [36]. Also, genes for resistance when introgressed from alien species are frequently diluted in its effectiveness in the hexaploid wheat background or are completely suppressed [37]—[41]. Marker assisted introgression has been shown as an effective approach for precise transfer of genes from wild species with minimum linkage drag [42] and also it could help in identifying genotypes containing the target gene in early generations even if it is suppressed in a particular genetic background [43]. Since the two PM resistance gene *PmTb7A*.*1* and *PmTb7A*.*2* identified in *T*. *boeoticum* pau5088 are located on the same chromosome arm (7AL) at a distance of ~48cM, the phenotype based selection in backcross progeny may not ensure transfer of the two genes independently but marker assisted alien introgression can ensure the transfer of the target genes with minimum linkage drag. Here we report precise transfer of the two PM resistance genes *PmTb7A*.*1* and *PmTb7A*.*2* from *T*. *boeoticum* to *T*. *aestivum* independently and in combination with minimum linkage drag using marker assisted selection. In addition to PM resistance, the *T*. *boeoticum* pau5088 also carries stem rust resistance gene *Sr22* (Harbans Bariana—personal communication) which maps very close to *PmTb7A*.*1*. We used *Sr22* linked markers also to monitor presence of *Sr22* in the progeny. To the best of our knowledge this is the first example of marker assisted transfer of an agronomically important gene from wild species to cultivated wheat.

## Materials and Methods

### Plant material

The plant material used in this study comprised PM resistant *Triticum boeoticum* (2n = 2X = 14) pau5088, *Triticum durum* cv. PBW114 (2n = 4X = 28) as bridging species, PBW343 introgression line (PBW343-IL) and PBW621. The PBW343-IL was generated by crossing PBW343 with a recombinant inbred line (RIL) derived from a cross of *T*. *boeoticum* acc pau5088/*T*. *monococcum* acc pau14087 [30] and is resistant to stripe and leaf rusts but susceptible to PM. Details of the PBW343-IL were presented in Chhuneja et al [40]. PBW621 is a recently released high yielding cultivar but it is susceptible to PM. *Triticum boeoticum* pau5088 is resistant to PM and the resistance is conferred by two genes, a novel gene and a new allele of *Pm1*, both tentatively designated as *PmTb7A*.*1* and *PmTb7A*.*2*, respectively [33], [34]. The *PmTb7A*.*1* is located about 48cM proximal to *PmTbA*.*2* and both the genes confer resistance to PM independently.

### Transfer of PM resistance to hexaploid wheat

**Fig 1:**
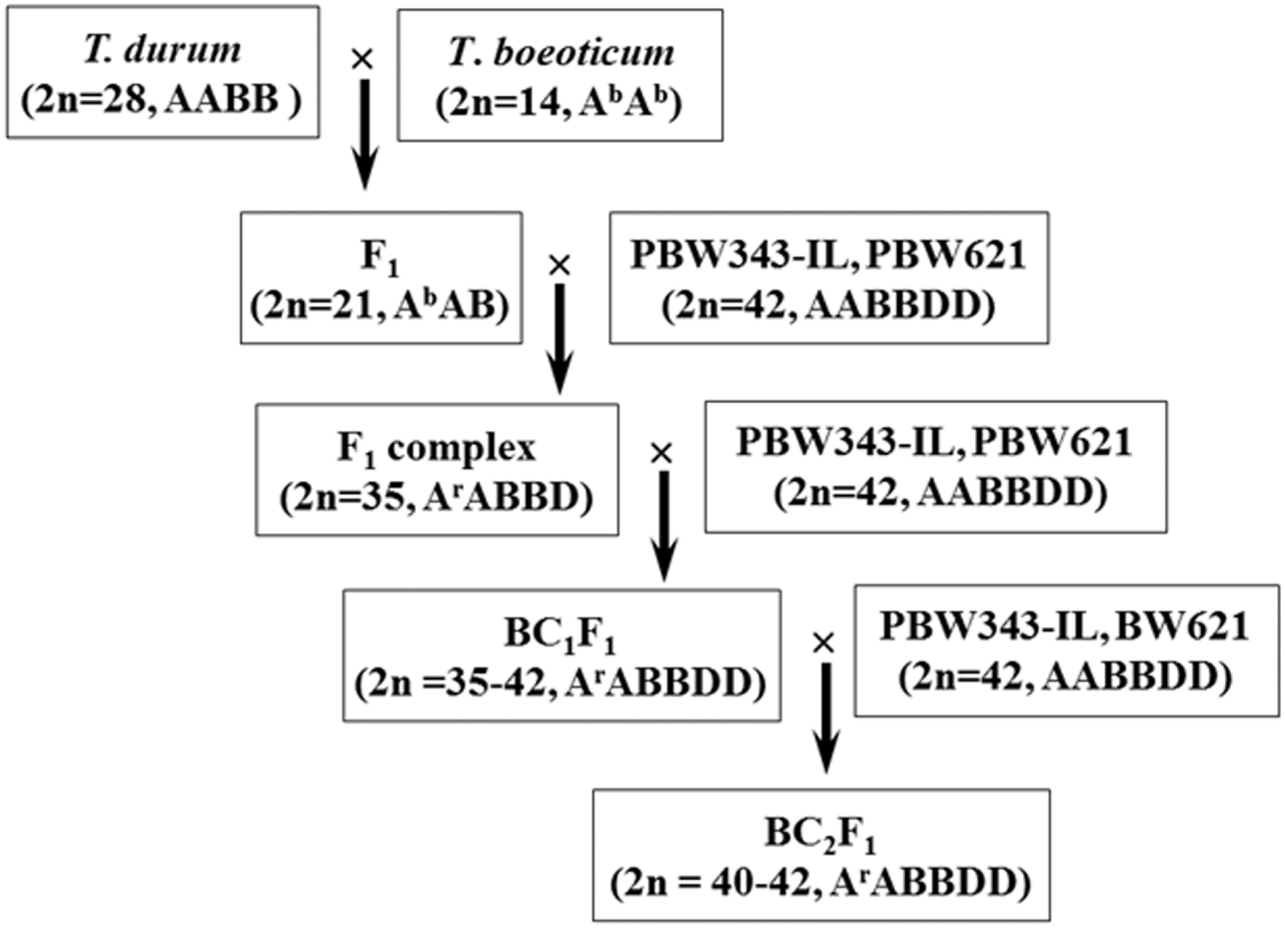
Schematic representation of the crossing strategy adopted for transferring powdery mildew resistance genes from *T*. *boeoticum* to hexaploid wheat *T*. *aestivum* cv. PBW343-IL and PBW621 using durum wheat as bridging species.

The PM resistant *T*. *boeoticum* acc 5088 (2n = 14, A^b^A^b^) was crossed as male to *T*. *durum* cv PBW114 (2n = 28, AABB). The F_1_ plants (2n = 21, A^b^AB) were crossed to hexaploid wheat genotypes PBW343-IL and PBW621 (2n = 42, AABBDD). The triploid F_1_ plants have both male as well as female sterility and only those gametes are viable which have near complete A and B genome chromosome complement [40]. Nearly 7000 florets of the F_1_ of the cross *T*. *durum* cv PBW 114/*T*. *boeoticum* pau5088 were pollinated with PBW 343-IL and 5300 florets pollinated with PBW621 and only 61 (0.87%) seeds were obtained after crossing with PBW343-IL and 65 (1.22%) seeds with PBW 621. resulting complex F_1_ plants primarily pentaploids (2n = 35, AABBD) have a D genome from hexaploid wheat, the B genome from both tetraploid and hexaploid wheat and the A genome from all the three species (Fig 1). These pentaploid F_1_ plants were expected to segregate for the target trait; PM resistance. The pentaploid F_1_ plants were analyzed with the markers flanking the PM resistance genes. The plants having introgression from *T*. *boeoticum* for the target markers were identified and backcrossed to hexaploid recurrent parent (RP). The selected BC_1_F_1_ progeny were planted during off-season at Keylong, Himachal Pradesh, India. These progeny were having varying chromosome number, ranging from 35–42, with modal class of 40–42. The BC_1_F_1_ plants were backcrossed to the RP to generate BC_2_F_1_, which were selfed to produce BC_2_F_2_ families from which homozygous resistant plants were selected (Fig 1).

### Screening against PM and stripe rust

PM appears naturally under field conditions at Ludhiana as well as at Keylong locations in India. In the experimental plots, susceptible check line PBW 343 was planted all around the plot and also after every 20 rows to ensure uniform spread of the disease in the field. Data on PM was recorded when disease score of the susceptible check line reached 8/9. Disease score of individual plants was recorded on 0–9 scale [44]—[46], with zero as immune, 1–3 as resistant, 4–6 as moderately resistant and 7–9 as susceptible. In all generations viz. complex F_1_, BC_1_F_1_, BC_2_F_1_ and parental lines were screened for two diseases: PM and stripe rust, for three consecutive crop seasons 2011–12, 2012–13 and 2013–14. Disease reaction was recorded on single plants, three times during the season at the adult plant stages; first week of March, last week of March and first week of April. Stripe rust, caused by *Puccinia striiformis* is a wide spread foliar disease in most wheat growing regions of the world including parts of India. *T*. *boeoticum* pau5088 and PBW343-IL were resistant to stripe rust [40], hence the progenies were screened for resistance to stripe rust also. Stripe rust severity was recorded on individual plants following modified Cobb’s scale [47] that includes disease severity (percentage of leaf area covered with rust urediospores) as well as disease response (infection type). The infection types were recorded as zero (immune); TR (traces of severity); MR (moderately resistant), MS (moderately susceptible); S (susceptible) and disease severity was recorded as percent leaf area infected.

### DNA extraction and marker analysis

Genomic DNA was isolated from parental lines *T*. *boeoticum* pau5088, *T*. *durum* PBW114, PBW343-IL and PBW621 and individual plants from various segregation generations following CTAB (Cetyl trimethyl ammonium bromide) method as modified by Allen et al. [48]. PCR conditions for RGA-STS markers linked to the target PM resistance genes were the same as reported in Chhuneja et al. [34].

### Marker assisted foreground selection

Two markers *Xwmc633* and *7AL-4556232_rga* flanking PM resistance gene *PmTb7A*.*1* and two markers*7AL-4426363_rga* and *7AL-4544237_rga* linked to *PmTbA*.*2* (Table 1) were used for foreground selection. The F_1_, complex F_1_, BC_1_F_1_ and BC_2_F_1_ were screened with these four markers. The PCR products were resolved in 6.0% non-denaturing polyacrylamide gels for SSR marker *Xwmc633* and 1.5% agarose gel for RGA-STS markers *7AL-4556232_rga*, *7AL-4426363_rga and 7AL-4544237_rga*. *7AL-4426363_rga* and *7AL-4544237_rga* were mapped as cleaved amplification polymorphism system (CAPS) markers. The amplified products were digested with 1U of *Taq1* and *Hph1* restriction enzymes, respectively.

**Table 1:** Primer sequences and annealing temperature of the linked markers used for transfer of *PmTb7A*.*1* and *PmTb7A*.*2* from *T*. *boeoticum* to bread wheat.

Gene	Linked marker	Primer Sequence (5’-------- 3’)	Annealing temperature (°C)
***PmTb7A.1***	*Xwmc633* F	ACACCAGCGGGGATATTTGTTAC	61
	*Xwmc633* R	GTGCACAAGACATGAGGTGGATT	
	*7AL-4556232_rga* F	TTTCAAATAACGGCTTCTGG	55
	*7AL-4556232_rga* R	GAGACGAGCAAATAGATATGG	
***PmTb7A.2***	*7AL-4426363_rga* F	GAATCCTCCAAAGCCTCCAC	60
	*7AL-4426363_rga* R	GGCATATCTCATGTGAAGAACTG	
	*7AL-4544237_rga* F	CACTACAATGATGGTAAGCGA	55
	*7AL-4544237_rga* R	GCAAGAAGAAACAAGGAGAG	

### Amplification of *Sr22* specific marker

Primer pair csIH81-BM (Forward 5’- TTCCATAAGTTCCTACAGTAC -3’; Reverse-5’- TAGACAAACAAGATTTAGCAC -3’) was used to amplify a DNA sequence specific for *Sr22* carrying segments of *T*. *boeoticum*, whereas primer pair csIH81-AG (Forward-5’- CTACCTCTGTCAATTTGAAC -3’; Reverse-5- GAAAAATGACTGTGATCGC -3’) was used to amplify corresponding fragments from genotypes lacking the *Sr22* carrying introgression [36]. In order to optimize multiplex PCR conditions for use as a co-dominant marker assay, 10μM concentration stocks of primers csIH81-BM and csIH81-AG were mixed in volume ratios (BM: AG) 1μl: 0.5 μl. Thermal cycling conditions included: 94°C for 5 min followed by 34 cycles of 94°C (denaturation) for 60s, 58°C (annealing) for 60s, 72°C (elongation) for 60s, followed by an elongation step of 7 min at 72°C. Amplification was tested by resolving PCR products in 1.5% agarose gel.

### Marker assisted background selection

For recurrent parent genome recovery, the background selection for the carrier chromosome was carried out in the BC_2_F_1_ generation. Forty SSR and 10 RGA markers, distributed uniformly throughout chromosome 7A, were screened for polymorphism among the diploid, the tetraploid and the hexaploid parental lines. Out of the 50 markers tested 16 SSR and 5 RGA markers that were polymorphic between donor parent *T*. *boeoticum* and the recipient parental lines PBW343-IL, PBW114 and PBW621 (S1 Table) were used for background selection. Details of the PCR conditions and map locations of these markers are available in Chhuneja et al. [34]. The recurrent parent genome recovery in the elite selections was calculated and graphically represented using the software Graphical Genotypes (GGT) Version 2.0 [49].

## Results

### Transfer of PM resistance

**Fig 2:**
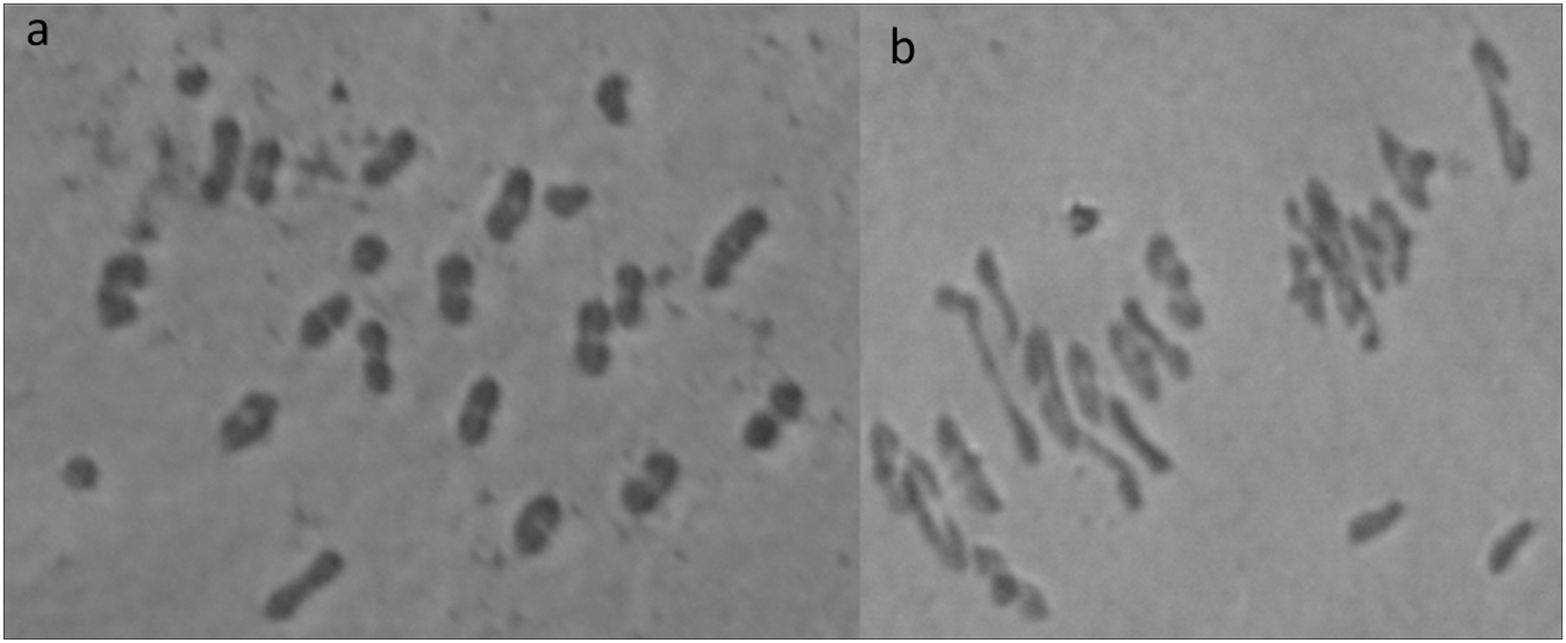
Meiotic analysis in selected BC_2_F_1_ plants a) PBW114/*T*. *boeoticum* pau5088 //3*PBW343-IL with 2n = 40 (18''+4'), b) PBW114/*T*. *boeoticum* pau5088//3*PBW621 with 2n = 42 (19''+4').

The breeding strategy for the transfer of the PM resistance genes from *T*. *boeoticum* followed in the present study is presented in Fig 1. The F_1_ plants of the cross *T*. *durum* cv PBW114/*T*. *boeoticum* were vigorous but completely male sterile. A total of 12,317 florets from 14 F_1_ plants were pollinated either with PBW343-IL or PBW621 and 126 pentaploid F_1_ seeds were generated. However, only 78 pentaploid F_1_ seeds germinated and survived in the field, which were later backcrossed to the respective recurrent parent (Table 2). Selected BC_1_F_1_ plants from a total of 239 were backcrossed to recurrent parents to generate BC_2_F_1_. Out of a total of 527 BC_2_F_1_ plants, 214 plants were used for marker analysis. All the selected BC_2_F_1_ plants were backcrossed as well as selfed to generate BC_3_F_1_ and BC_2_F_2_, respectively. Chromosome number in the selected BC_2_F_1_ plants varied from 40–42 and number of univalents varied from 2–4 (Fig 2).

**Table 2:** Summary of the material generated for transfer of *PmTb7A*.*1* and *PmTbA*.*2* from *T*. *boeoticum* to hexaploid wheat.

Cross	Generation and year	Total seeds obtained	Total plants survived
**PBW114/*T*. *boeoticum* pau5088**	F_1_ (main campus- 2011/12)	24	14
**PBW114/*T*. *boeoticum* pau5088// PBW343-IL**	Complex F_1_ (main season- 2012/13)	61	36
**PBW114/*T*. *boeoticum* pau5088// PBW621**	Complex F_1_ (main season- 2012/13)	65	42
PBW114/*T*. *boeoticum* pau5088// 2*PBW343-IL	BC_1_F_1_ (offseason -2013)	1756	118
PBW114/*T*. *boeoticum* pau5088// 2*PBW621	BC_1_F_1_ (offseason -2013)	1316	121
PBW114/*T*. *boeoticum* pau5088// 3*PBW343-IL	BC_2_F_1_ (main season- 2013/14)	752	282
PBW114/*T*. *boeoticum* pau5088// 3*PBW621	BC_2_F_1_ (main season- 2013/14)	639	245

### Foreground selection

**Fig 3:**
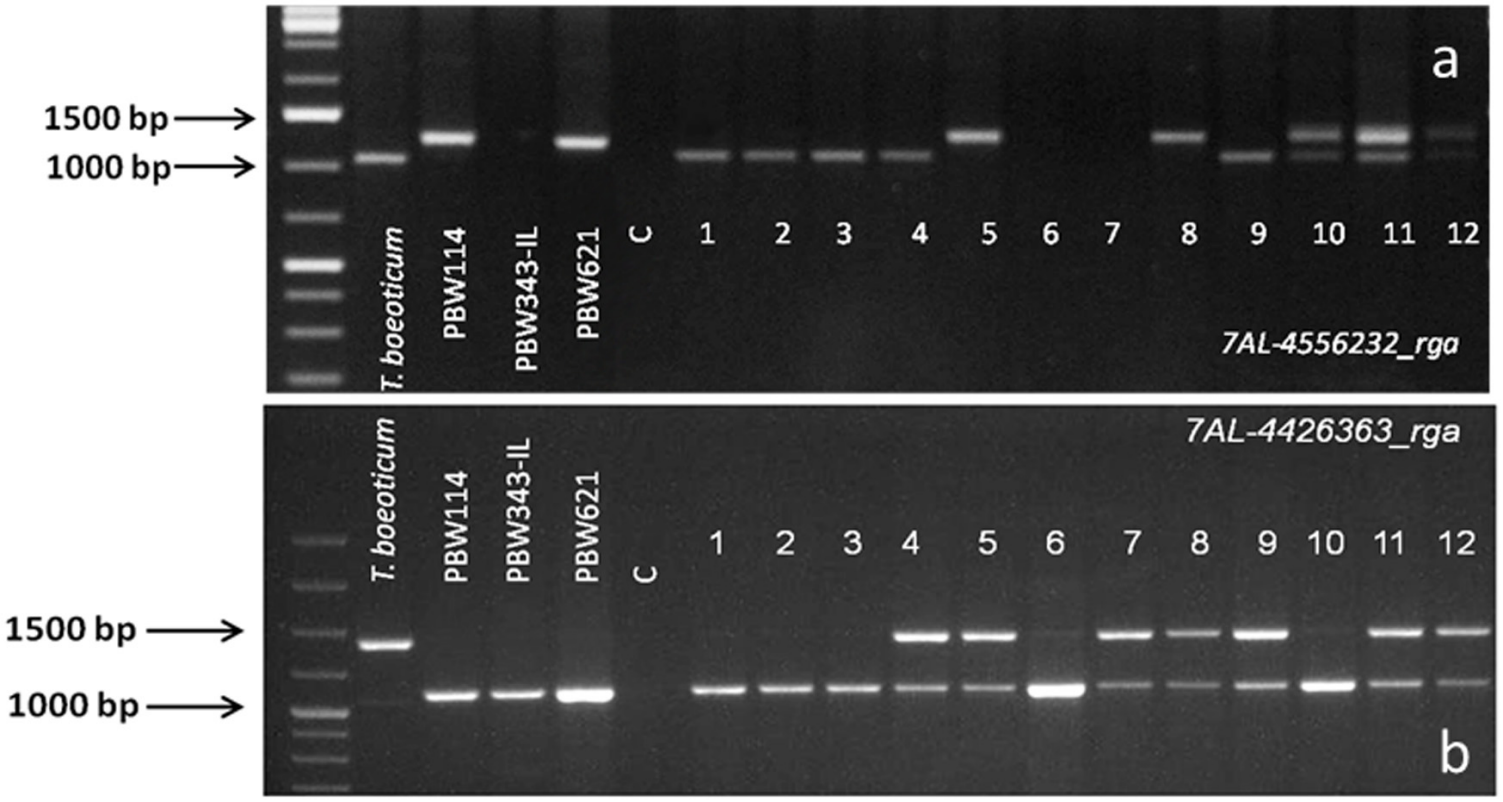
*In vitro* amplification profile of RGA-STS markers linked to *PmTb7A*.1and *PmTb7A*.*2* a) *7AL-4556232_rga*, b) *7AL-4426363_rga*. Numbers 1–12 represent different BC_2_F_1_ plants with either one or both the genes.

Two flanking markers for each of the two PM resistance genes were used for foreground selection. For *PmTb7A*.*1*, *Xwmc633* and *7AL-4556232_rga* and for *PmTb7A*.*2*, *7AL-4426363_rga* and *7AL-4544237_rga* were used (Fig 3). The F_1_ pentaploid (2n = 35, AABBD), BC_1_F_1_ and BC_2_F_1_ plants were genotyped using the four flanking markers for two PM resistance genes *PmTb7A*.*1* and *PmTb7A*.*2*. Details of the population size and marker analysis for foreground selection in the two cross combinations involving recipient parents PBW343-IL and PBW621 is presented in Table 3. In BC_2_F_1_, phenotypic selections were practiced based on agromorphological traits of the plants positive for *PmTb7A*.*1* and/or *PmTb7A*.*2* and a total of 40 agronomically desirable plants were selected for carrying forward and for assessing the recurrent parent genotype recovery.

**Table 3:** Marker analysis for the powdery mildew resistance genes *PmTb7A*.*1* and *PmTbA*.*2* in pentaploid F_1_, BC_1_F_1_ and BC_2_F_1_.

Generation	Total plantsanalysed	No. of plants positive for *T*. *boeoticum* allele(s) of		
		*PmTb7A*.*1*	*PmTb7A*.*2*	*PmTb7A*.*1 + PmTb7A*.*2*
**PBW114/*T*. *boeoticum*//PBW343-IL**				
F_1_ pentaploid	36	11	3	9
BC_1_F_1_	117	34	5	25
BC_2_F_1_	121	41	13	6
**PBW114/*T*. *boeoticum*//PBW621**				
Pentaploid F_1_	42	8	7	21
BC_1_F_1_	98	22	26	21
BC_2_F_1_	93	21	16	14
**Total plants**				
Pentaploid F_1_	78	19	10	30
BC_1_F_1_	215	56	31	46
BC_2_F_1_	214	68	29	20

### Phenotypic evaluation for PM and stripe rust resistance

**Fig 4:**
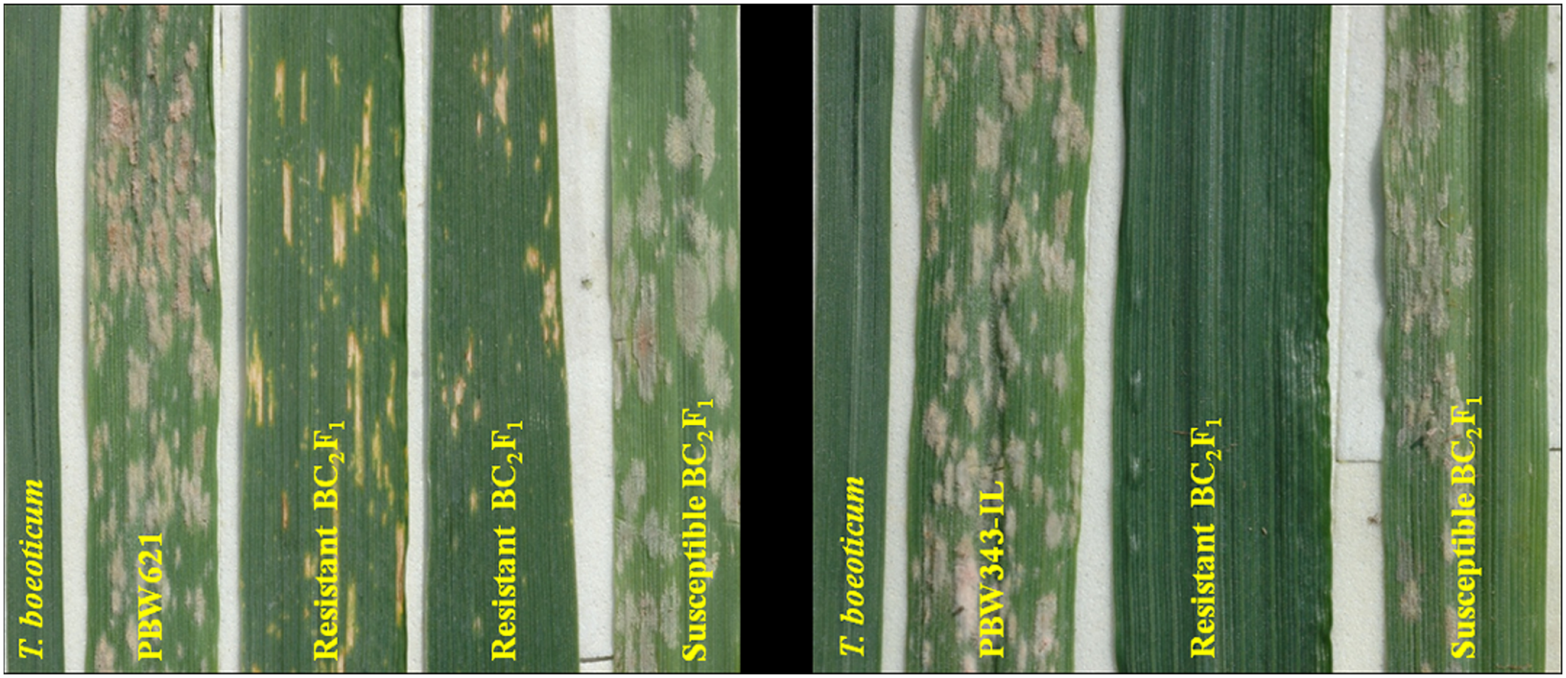
Powdery mildew reaction of the parents and introgression lines developed from the cross a) *T*. *durum* cv. PBW114*/ T*. *boeoticum* pau5088 //3*PBW343-IL, b) *T*. *durum* cv. PBW114*/ T*. *boeoticum* pau5088 //3*PBW621 at the adult plant stage under field conditions.

At adult plant stage (APS), *T*. *boeoticum* pau5088 was resistant with no traces of disease, while *T*. *durum* cv PBW114, PBW343-IL and PBW621 recorded PM score of 8–9 (Fig 4). Out of the 121 BC_2_F_1_ plants from the cross PBW114/*T*. *boeoticum*//3*PBW343-IL, 66 were resistant and 55 susceptible (Table 4) whereas out of 93 BC_2_F_1_ plants from the cross PBW114/*T*. *boeoticum*//3*PBW621, 51 were resistant and 42 susceptible (Table 4). PM reaction of the representative plants is shown in Fig 4 and detailed in Table 5. All the plants positive for the markers flanking *PmTb7A*.*1* and/or *PmTb7A*.*2* were resistant to PM at the adult plant stage indicating that both the genes were effective individually also.

**Table 4:** Frequency of powdery mildew resistant BC_2_F_1_ plants with different gene combinations stage.

Cross	Gene combination			Total plants screened
	*PmTb7A*.*1*	*PmTb7A*.*2*	*PmTb7A*.*1 + PmTb7A*.*2*	
PBW114/ *T*. *boeoticum* //3*PBW343-IL	41 (37)^a^	13 (9)	6 (4)	121
PBW114/ *T*. *boeoticum* //3*PBW621	21 (20)	16 (15)	14 (10)	93

^a^ Numbers in parentheses indicate the number of plants that were resistant to stripe rust also.

**Table 5:** Frequency of BC_2_F_1_ plants with varying levels of powdery mildew score at adult plant stage under field conditions during 2014.

Disease reaction	Number of plants in the cross	
	PBW114/ *T*. *boeoticum* acc. pau5088//3*PBW343-IL	PBW114/ *T*. *boeoticum* acc. pau5088//3*PBW621
**0**	**51** ^a^	**46**
**1**	**0**	**0**
**2**	**2**	**2**
**3**	7	3
**4**	14	7
**5**	5	8
**6**	8	7
**7**	9	2
**8**	15	10
**9**	10	8
**Total**	121	93

^a^ Numbers in bold are the number of BC_2_F_1_ plants resistant to PM and carrying different gene combinations as detailed in Table 4.

All the PM resistant plants were also screened for stripe rust resistance as we expected segregation in for this trait due to presence of suppressor gene from *T*. *durum*. Both the donor parent and the recurrent parent PBW343-IL were resistant to stripe rust but the BC_2_F_1_ population segregated for stripe rust resistance. Out of the 121 BC_2_F_1_ plants from the cross PBW114/*T*. *boeoticum*//3*PBW343-IL, 87 plants were resistant to stripe rust and 34 were susceptible. However, out of 60 PM resistant plants, 41 were resistant to stripe rust as well (S2 Table). Similarly, out of 93 BC_2_F_1_ plants from the cross PBW114/*T*. *boeoticum*//3*PBW621, 74 were resistant and 19 susceptible for stripe rust but among the 51 PM resistant plants, 39 were resistant to stripe rust also (S3 Table).

### Screening for molecular markers linked to stem rust resistance gene *Sr22*

**Fig 5:**
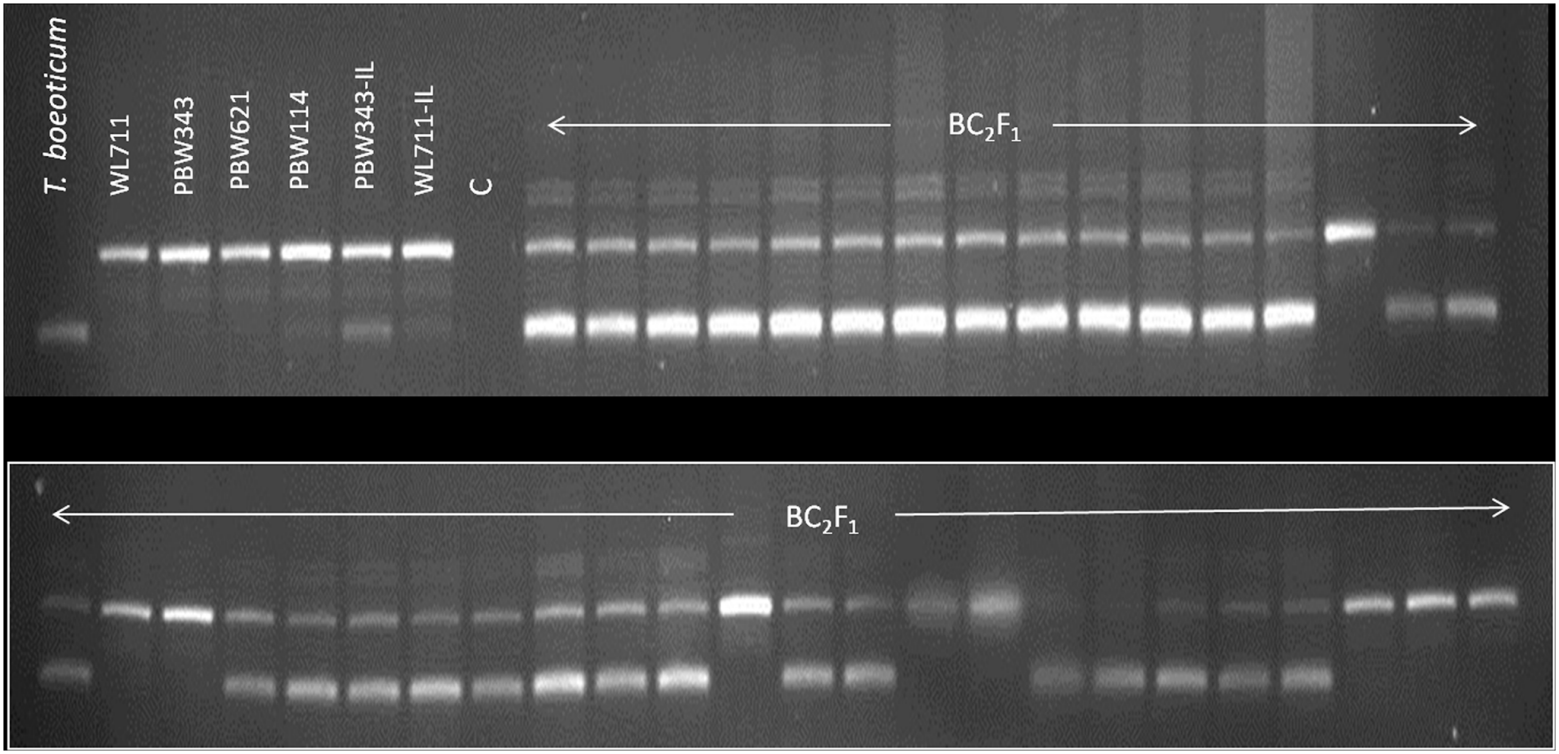
Amplification profile of the 40 selected BC_2_F_1_ plants carrying *PmTb7A*.*1* and/or *TmTb7A*.*2* with *Sr22* specific marker *Xsr22*: *XcsIH81-BM/ XcsIH81-AG*.

The stem rust resistance gene *Sr22*, derived from *Triticum boeoticum* acc G-21 [50] and *T*. *monococcum* acc. RL5244 [51] confers resistance to *Puccinia graminis* f. sp. *tritici* race TTKSK (also known as Ug99) [35]. Despite the A genome of *T*. *boeoticum* having close homology to the A genome of *T*. *aestivum*, the *Sr22* carrying lines are agronomically poor [35]. *Xcfa2123*, *Xwmc633* and *cssu22* have been reported as the most tightly linked proximal and distal SSR markers, respectively, to the *Sr22* gene [35], [36]. *Xwmc633* is also closely linked to PM resistance gene *PmTb7A* [34]. *T*. *boeoticum* pau5088 is resistant to stem rust race Ug99 (Harbans Bariana, personal communication) and it showed the presence of the *Sr22* allele when analyzed with markers closely linked to the gene. So the selected BC_2_F_1_ plants were also analysed with the *Sr22* linked marker to detect if the *Sr22* has been co-introgressed with *PmTb7A*.*1*. Among the 40 selected BC_2_F_1_ plants, 31 plants were heterozygous for the *Sr22* linked maker indicating the presence of the *Sr22* allele and 9 plants did not amplify *Sr22* specific allele (Fig 5). Of the nine plants lacking *Sr22* allele seven did not carry *PmTb7A*.*1* thereby indicating that only two BC_2_F_1_ plants were recombinants between *PmTb7A*.*1* and *Sr22* allele transferred from *T*. *boeoticum*.

### Introgression profiling of chromosome 7A

**Fig 6:**
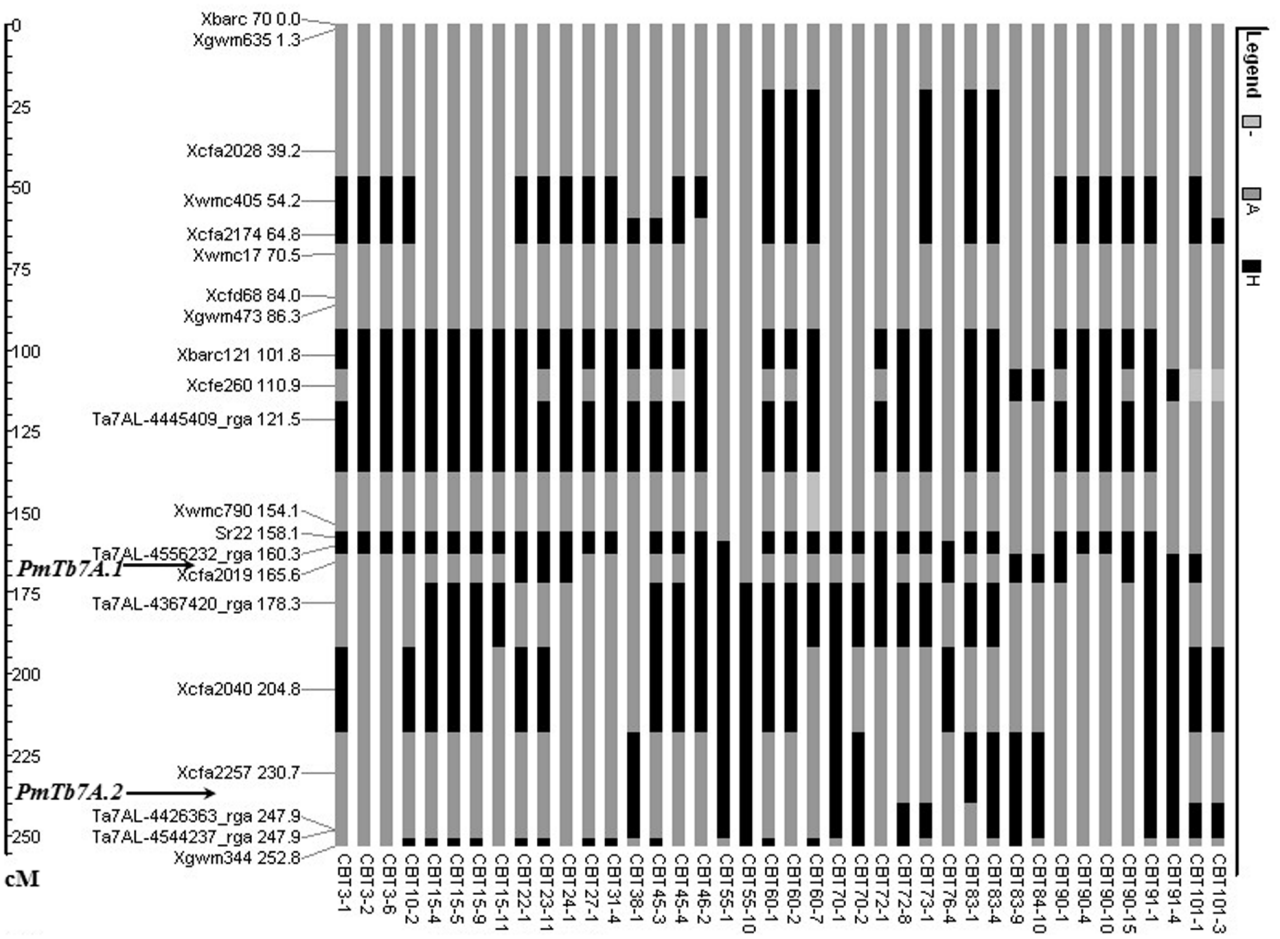
Introgression profile of selected BC_2_F_1_ plants for chromosome 7AL using SSR markers and gene based marker. Black area indicates *T*. *boeoticum* specific introgression and grey areas indicate recurrent parent genome. The chromosomal locations of various markers are as per Chhuneja et al. [34].

For analysing the marker profile of the introgression lines, the parental lines were analysed for polymorphism with 40 SSR and 10 RGA-STS markers. Only 20 markers were polymorphic between the donor and the recipient lines. The 40 PM resistant BC_2_F_1_ plants having either *PmTb7A*.*1* or *PmTbA*.*2* or both were analysed using SSR and gene based markers distributed throughout the carrier chromosome 7A to identify the plants having *PmTb7A*.*1* and *PmTbA*.*2* with minimum alien introgression. Introgression in the BC_2_F_1_ plants varied from 15.4–62.9% with minimum introgression in plant CBT76-4 which had *PmTb7A*.*1* only but not *PmTbA*.*2* and CBT101-3 which had introgression for *PmTbA*.*2* only and not for *PmTb7A*.*1*. However, both these plants had a common introgression around the marker region *Xcfa2040* (Fig 6). *T*. *boeoticum* introgressed segments varied from one in CBT55-10 to a maximum of six in CBT60-1 (Fig 6). The BC_2_F_1_ plants CBT76-4 and CBT101-3 were selfed and plants homozygous for *PmTb7A*.*1* and *PmTb7A*.*2* were selected for use as donors for the mobilization of *PmTb7A*.*1* and *PmTb7A*.*2* to other elite backgrounds. True breeding progeny of the plants CBT76-4 and CBT101-3 are assigned the accession numbers acc. pau16053 and acc. pau16054, respectively. Likewise, plants CBT3-2, CBT3-6, CBT27-1, and CBT31-4, all showed introgression for markers linked to PM resistance gene *PmTb7A*.*1*. These plants will also be crossed to plant CBT101-3 and recombinants having marker alleles linked to *PmTb7A*.*1* and *PmTb7A*.*2* from *T*. *boeoticum* but no introgression around the marker *Xcfa2040* present in plant CBT101-3 will be selected so as to have the plants with minimum amount of alien introgression.

## Discussion

PM and rusts are the most important foliar diseases of wheat and these have been contained primarily through resistance breeding. However, cyclic breakdown of the resistance genes demands for constant search of new genes. Pyramiding of two or more genes can help in increasing the life span of the genes when deployed over larger areas. But pyramiding of two or more resistance genes is possible only if closely linked DNA markers are available. Variability for disease resistance genes in cultivated germplasm is lower than in wild species germplasm; however, the transfer of the genes from secondary and tertiary genes pools is difficult and it is also associated with linkage drag, thus limiting their usefulness in commercializing these genes. With the advent of DNA markers it has now become possible to precisely transfer the desirable genes from unadapted germplasm to elite lines with minimum or no linkage drag [42]. Of the 193 designated genes for resistance to leaf rust, stripe rust, stem rust, PM and cereal cyst nematode in wheat, as many as 101 genes have been transferred from wild species but all of these could not be deployed in cultivars primarily due to associated linkage drag that affects yield and/or quality [52]. The primary gene pool of common wheat, including the A genome donor *T*. *urartu* and its relatives *T*. *monococcum* and *T*. *boeoticum*, is an important resource for useful variability for many economically important genes, including resistance to diseases [27]—[30]. Many of the PM resistance genes have been mapped on the 7AL chromosome of wheat including *Pm1*, *Pm9*, *Pm37* [31]—[32], [53]—[54] and many temporarily designated genes *mlRD30*, *PmU*, *Mlm2033*, *Mlm80*, *mlIW72*, *MlWE18*, *MlAG12*, *PmG16* [10], [39], [54]—[59].

Chhuneja et al. [40], while attempting to transfer stripe rust resistance genes from *T*. *monococcum* and a RIL derived from cross between *T*. *boeoticum*/*T*. *monococcum* had to backcross large number of pentaploid F_1_ plants to *T*. *aestivum* as none of the 225 plants exhibited resistance to stripe rust. Likewise, in one cross, only one BC_1_F_1_ plant out of 25 plants was resistant to stripe rust, thus limiting the choice for backcrossing. However, in another cross, 15 out of the 111 BC_1_F_1_ plants showed resistance to stripe rust. In the present study, out of 78 pentaploid F_1_ plants analyzed with markers as many as 59 plants had either one or both the PM resistance genes. Even if there was any suppression of the resistance it would not limit the choice of the plants for further backcrossing. Similarly, in the BC_1_F_1_ generation 133 out of 215 plants analyzed for markers had either one or both the PM resistance genes present. The approach of mapping desirable genes in wild species background and then transferring them using MAS may prove more useful than transferring these first in cultivated wheat background followed by mapping. However, both the approaches will have their own merits and limitations. *Sr22*, for example, transferred from *T*. *boeoticum* to hexaploid wheat has not been used widely because of linkage drag associated with it. As this gene confers resistance to stem rust race TTKSK (also known as Ug99), renewed interest in its deployment demanded shortening of the introgressed segments. DNA markers closely linked to *Sr22* were identified and used to shorten the introgressed segments [35], [36]. The approach of mapping the genes in wild species background followed by marker assisted transfer adopted in the present study has resulted into development of introgression lines which are agronomically as good as the recipient elite lines.

In the present study it is not clear whether the PM resistance suppression occurred during early generations or not as we did not create any artificial epiphytotic for PM but stripe rust resistance suppression did occur even in BC_2_F_1_ generations. The recipient line PBW343-IL was resistant to stripe rust and in BC_2_F_1_ one copy of the genome is contributed by the recurrent parent, thus all the BC_2_F_1_ plants were expected to be resistant to stripe rust. However, out of 60 BC_2_F_1_ plants from the cross PBW114/ *T*. *boeoticum* //3*PBW343-IL, found to be positive for markers linked to *PmTb7A*.*1* or *PmTbA*.*2*, only 44 plants were resistant to stripe rust and 14 were susceptible. This is not possible until suppressor genes were present in some progeny. It was also possible to transfer the two linked PM resistance genes independently with minimum alien introgression. The two lines will now be crossed to pyramid the two genes *PmTb7A*.*1* and *PmTb7A*.*2* into single genotype with minimum linkage drag through MAS. Our study provided complete strategy for transferring PM resistance genes from wild species to cultivated varieties with minimum linkage drag and maximum recovery of the recurrent parent genome. To the best of our knowledge it is the first example in wheat for marker assisted transfer of two related genes from wild species into cultivated wheat with minimum linkage drag. The resulting introgression lines have resistance to stripe rust, leaf rust, stem rust and PM all transferred from *T*. *boeoticum*.

## Supporting Information

S1 Table Nucleotide sequences of the primer pairs used for marker assisted background selection of carrier chromosome. (DOCX)

S2 Table Powdery mildew reaction and marker data of selected BC_2_F_1_ plants obtained from the cross of *T*. *durum* cv PBW114/*T*. *boeoticum* acc. Pau5088//3*PBW343-IL. (DOCX)

S3 Table Powdery mildew reaction and marker data of selected BC_2_F_1_ plants obtained from the cross of *T*. *durum* cv PBW114/*T*. *boeoticum* acc. Pau5088//3*PBW621. (DOCX)
