# Supplementary material for: Marker Assisted Transfer of Two Powdery Mildew Resistance Genes PmTb7A.1 and PmTb7A.2 from Triticum boeoticum (Boiss.) to Triticum aestivum (L.)
Source: PLoS One. 2015 Jun 11;10(6):e0128297. doi: 10.1371/journal.pone.0128297 (PMC4466026; doi:10.1371/journal.pone.0128297)
Supplement: S1 Table — (DOCX) [file pone.0128297.s001.docx]

Supplementary table S1. Nucleotide sequences of the primer pairs used for marker assisted background selection of carrier chromosome

| S. No | Marker name | Marker position^a^ | Primer sequence  (5’ ------- 3’) | Annealing temp. |
| --- | --- | --- | --- | --- |
| 1 | *Xbarc70* | 0 | F- GCGAAAAACGATGCGACTCAAAG  R- GCGCCATATAATTCAGACCCACAAAA | 55°C |
| 2 | *Xgwm635* | 1.3 | F- TTCCTCACTGTAAGGGCGTT  R- CAGCCTTAGCCTTGGCG | 60°C |
| 3 | *Xcfa2028* | 39.2 | F- TGGGTATGAAAGGCTGAAGG  R- ATCGCGACTATTCAACGCTT | 60°C |
| 4 | *Xwmc405* | 54.2 | F- GTGCGGAAAGAGACGAGGTT  R- TATGTCCACGTTGGCAGAGG | 61°C |
| 5 | *Xcfa2174* | 64.8 | F- ACGGCATCACAGGTTAAAGG  R- GTCTTTGCACTGCTAGCCT | 60°C |
| 6 | *Xwmc17* | 70.5 | F- 5`-ACCTGCAAGAAATTAGGAACTC  R- 5`-CTAGTGTTTCAAATATGTCGGA | 51°C |
| 7 | *Xcfd68* | 84 | F- TTTGCAGCATCACACGTTTT  R- AAATTGTATCCCCCGTGGT | 60°C |
| 8 | *Xgwm473* | 86.3 | F- TCATACGGGTATGGTTGGAC  R- CACCCCCTTGTTGGTCAC | 55°C |
| 9 | *Xbarc121* | 101.8 | F- ACTGATCAGCAATGTCAACTGAA  R- CCGGTGTCTTTCCTAACGCTATG | 50°C |
| 10 | *Xcfe260* | 110.9 | F- AAGCAGCTCCAAACACCAAG  R- CTCAGTGACAAGGACGACGA | 60°C |
| 11 | *7AL-4445409* | 121.5 | F- CTTACGTTCTTGGTCCTCAC  R- AGCTGAGAAACTCAAATCTG | 55°C |
| 12 | *Xwmc790* | 154.1 | F- ATTAAGATAGACCGTCCATATCATCCA  R- CGACAACGTACGCGCC | 61°C |
| 13 | *Sr22: XcsIH81-BM/ XcsIH81-AG* | 158.1 | F- TTCCATAAGTTCCTACAGTAC  R- TAGACAAACAAGATTTAGCAC | 58°C |
|  |  |  | F- CTACCTCTGTCAATTTGAAC  R- GAAAAATGACTGTGATCGC |  |
| 14 | *7AL-4426232* | 160.3 | F- TTTCAAATAACGGCTTCTGG  R- GAGACGAGCAAATAGATATGG | 55°C |
| 15 | *Xcfa2019* | 165.6 | F- ACGAGCTAACTGCAGACCC  R- CAATCCTGATGCGGAGAT | 60°C |
| 16 | *7AL-4367420* | 178.3 | F- GTGTAGATATGGTGTAGAGAAGAC  R- GTCAAACAATCCGAGGTAAACTG | 55°C |
| 17 | *Xcfa2040* | 204.8 | F- TCAAATGATTTCAGGTAACCACTA  R- TCCTGATCCCACCAAACAT | 60°C |
| 18 | *Xcfa2257* | 230.7 | F- GATACAATAGGTGCCTCCGC  R- CCATTATGTAAATGCTTCTGTTTGA | 60°C |
| 19 | *7AL-4426363* | 247.9 | F- GAATCCTCCAAAGCCTCCAC  R- GGCATATCTCATGTGAAGAACTG | 60°C |
| 20 | *7AL-544237* | 247.9 | F- CACTACAATGATGGTAAGCGA  R- GCAAGAAGAAACAAGGAGAG | 55°C |
| 21 | *Xgwm344* | 252.8 | F- CAAGGAAATAGGCGGTAACT  R- ATTTGAGTCTGAAGTTTGCA | 55°C |

^a^ Marker position is as per Chhuneja et al [34]
