# Supplementary material for: Marker Assisted Transfer of Two Powdery Mildew Resistance Genes PmTb7A.1 and PmTb7A.2 from Triticum boeoticum (Boiss.) to Triticum aestivum (L.)
Source: PLoS One. 2015 Jun 11;10(6):e0128297. doi: 10.1371/journal.pone.0128297 (PMC4466026; doi:10.1371/journal.pone.0128297)
Supplement: S2 Table — (DOCX) [file pone.0128297.s002.docx]

Supplementary Table S2. Powdery mildew reaction and marker data of selected BC_2_F_1_ plants obtained from the cross of *T. durum* cv PBW114/*T. boeoticum* acc. Pau5088//3*PBW343-IL ^a^

| S. No. | Plant ID | *PmTb7A.1* linked markers | | *PmTb7A.2* linked markers | | PM score | Stripe rust reaction |
| --- | --- | --- | --- | --- | --- | --- | --- |
|  |  | *7AL-4556232* | *Xwmc633* | *7AL-4426363* | *7AL-4544237* |  |  |
| 1 | CBT3-1 | **+^b^** | **+** | **-** | **-** | 0 | 10MR |
| 2 | CBT3-2 | **+** | **+** | **-** | **-** | 0 | 20MR |
| 3 | CBT3-3 | **+** | **+** | **-** | **-** | 0 | 10MR |
| 4 | CBT3-6 | **+** | **+** | **-** | **-** | 0 | 0 |
| 5 | CBT10-2 | **+** | **+** | **-** | **-** | 0 | 10MR |
| 6 | CBT15-4 | **+** | **+** | **-** | **-** | 3 | 10MR |
| 7 | CBT15-5 | **+** | **+** | **-** | **-** | 3 | 0 |
| 8 | CBT15-9 | **+** | **+** | **-** | **-** | 0 | 10MR |
| 9 | CBT15-11 | **+** | **+** | **-** | **-** | 0 | 10MR |
| 10 | CBT16-1 | **+** | **+** | **+** | **+** | 0 | 10MR |
| 11 | CBT22-1 | **+** | **+** | **-** | **-** | 0 | 0 |
| 12 | CBT22-2 | **+** | **+** | **-** | **-** | 0 | 5MR |
| 13 | CBT23-1 | **+** | **+** | **-** | **-** | 0 | 20S |
| 14 | CBT23-3 | - | - | + | + | 3 | 10MR |
| 15 | CBT23-11 | **+** | **+** | **-** | **-** | 0 | 10MR |
| 16 | CBT23-12 | **+** | **+** | **-** | **-** | 0 | 20MR |
| 17 | CBT24-1 | **+** | **+** | **-** | **-** | 3 | 5MR |
| 18 | CBT27-1 | **+** | **+** | **-** | **-** | 0 | 5MR |
| 19 | CBT27-2 | **+** | **+** | **-** | **-** | 0 | 10MR |
| 20 | CBT29-1 | **+** | **+** | **-** | **-** | 0 | 40S |
| 21 | CBT31-1 | **+** | **+** | **-** | **-** | 0 | 10MR |
| 22 | CBT31-3 | **+** | **+** | **-** | **-** | 0 | 5MR |
| 23 | CBT31-4 | **+** | **+** | **-** | **-** | 0 | 0 |
| 24 | CBT31-6 | **+** | **+** | **-** | **-** | 0 | 20S |
| 25 | CBT32-1 | **+** | **+** | **-** | **-** | 0 | 20S |
| 26 | CBT32-2 | **+** | **+** | **-** | **-** | 0 | 40S |
| 27 | CBT32-3 | **+** | **+** | **-** | **-** | 0 | 20MR |
| 28 | CBT34-1 | **+** | **+** | **-** | **-** | 0 | 0 |
| 29 | CBT37 | **-** | **-** | **+** | **+** | 0 | 0 |
| 30 | CBT38-1 | **-** | **-** | **+** | **+** | 0 | 20S |
| 31 | CBT38-3 | **-** | **-** | **+** | **+** | 0 | 20MR |
| 32 | CBT42 | **-** | **-** | **+** | **+** | 2 | 40S |
| 33 | CBT45-3 | **+** | **+** | **-** | **-** | 0 | 20MR |
| 34 | CBT45-4 | **+** | **+** | **-** | **-** | 0 | 10MR |
| 35 | CBT45-7 | **+** | **+** | **-** | **-** | 0 | 60S |
| 36 | CBT45-8 | **+** | **+** | **-** | **-** | 0 | 20S |
| 37 | CBT46-1 | **+** | **+** | **-** | **-** | 3 | 5MR |
| 38 | CBT46-2 | **+** | **+** | **-** | **-** | 2 | 0 |
| 39 | CBT46-3 | **+** | **+** | **-** | **-** | 0 | 5MR |
| 40 | CBT53-1 | **-** | **-** | **+** | **+** | 0 | 20S |
| 41 | CBT53-2 | **+** | **+** | **+** | **+** | 0 | 0 |
| 42 | CBT53-3 | **+** | **+** | **-** | **-** | 0 | 20MR |
| 43 | CBT54-4 | **-** | **-** | **+** | **+** | 0 | 0 |
| 44 | CBT54-5 | **-** | **-** | **+** | **+** | 0 | 0 |
| 45 | CBT54-6 | **-** | **-** | **+** | **+** | 0 | 0 |
| 46 | CBT54-9 | **-** | **-** | **+** | **+** | 0 | 0 |
| 47 | CBT54-10 | **+** | **-** | **-** | **+** | 3 | 0 |
| 48 | CBT54-12 | **-** | **-** | **+** | **+** | 3 | 0 |
| 49 | CBT54-13 | **-** | **-** | **+** | **+** | 0 | 0 |
| 50 | CBT55-1 | **+** | **+** | **+** | **+** | 0 | 0 |
| 51 | CBT55-2 | **-** | **-** | **+** | **+** | 0 | 10MR |
| 52 | CBT55-4 | **+** | **+** | **-** | **-** | 0 | 0 |
| 53 | CBT55-5 | **+** | **+** | **+** | **+** | 0 | 20MR |
| 54 | CBT55-6 | **+** | **+** | **-** | **-** | 0 | 0 |
| 55 | CBT55-7 | **+** | **+** | **-** | **-** | 0 | 0 |
| 56 | CBT55-8 | **+** | **+** | **+** | **+** | 0 | 20S |
| 57 | CBT55-9 | **+** | **+** | **-** | **-** | 0 | 10MR |
| 58 | CBT55-10 | **-** | **-** | **+** | **+** | 0 |  |
| 59 | CBT55-11 | **+** | **+** | **-** | **-** | 0 |  |
| 60 | CBT55-12 | **+** | **+** | **+** | **+** | 0 |  |

^a^ PBW 343-IL in hexaploid wheat *T. aestivum;* ^b^ ‘+’ and ‘-‘ denote the presence and absence of desirable allele of the gene
