# Supplementary material for: Marker Assisted Transfer of Two Powdery Mildew Resistance Genes PmTb7A.1 and PmTb7A.2 from Triticum boeoticum (Boiss.) to Triticum aestivum (L.)
Source: PLoS One. 2015 Jun 11;10(6):e0128297. doi: 10.1371/journal.pone.0128297 (PMC4466026; doi:10.1371/journal.pone.0128297)
Supplement: S3 Table — (DOCX) [file pone.0128297.s003.docx]

Supplementary Table S3. Powdery mildew reaction and marker data of selected BC_2_F_1_ plants obtained from the cross of *T. durum* cv PBW114/*T. boeoticum* acc. Pau5088//3*PBW621^a^

| S. No. | Plant ID | *PmTb7A.1* linked markers | | *PmTb7A.2* linked markers | | PM score | Stripe rust reaction |
| --- | --- | --- | --- | --- | --- | --- | --- |
|  |  | *7AL-4556232* | *Xwmc633* | *7AL-4426363* | *7AL-4544237* |  |  |
| 1 | CBT60-1 | **+^b^** | **+** | **-** | **-** | 0 | 0 |
| 2 | CBT60-2 | **+** | **+** | **-** | **-** | 0 | 5MR |
| 3 | CBT60-4 | **+** | **+** | **-** | **-** | 0 | 0 |
| 4 | CBT60-5 | **+** | **+** | **+** | **+** | 0 | 10MR |
| 5 | CBT60-7 | **+** | **+** | **-** | **-** | 0 | 0 |
| 6 | CBT64-1 | **-** | **-** | **+** | **+** | 0 | 0 |
| 7 | CBT64-2 | **-** | **-** | **+** | **+** | 0 | 5MR |
| 8 | CBT64-3 | **-** | **-** | **+** | **+** | 0 | 20MR |
| 9 | CBT64-4 | **-** | **-** | **+** | **+** | 0 | 20MR |
| 10 | CBT64-5 | **-** | **-** | **+** | **+** | 0 | 10MR |
| 11 | CBT70-1 | **+** | **+** | **+** | **+** | 0 | 0 |
| 12 | CBT70-2 | **+** | **+** | **+** | **+** | 0 | 0 |
| 13 | CBT70-3 | **+** | **+** | **+** | **+** | 0 | 20MR |
| 14 | CBT70-4 | **+** | **+** | **+** | **+** | 2 | 20S |
| 15 | CBT72-1 | **+** | **+** | **-** | **-** | 0 | 10M |
| 16 | CBT72-2 | **+** | **+** | **+** | **+** | 0 | 20MR |
| 17 | CBT72-5 | **-** | **-** | **+** | **+** | 0 | 0 |
| 18 | CBT72-8 | **+** | **+** | **+** | **+** | 0 | 0 |
| 19 | CBT73-1 | **+** | **+** | **+** | **+** | 0 | 0 |
| 20 | CBT73-5 | **+** | **+** | **+** | **+** | 0 | 5MR |
| 21 | CBT76-4 | **+** | **+** | **-** | **-** | 3 | 0 |
| 22 | CBT83-1 | **+** | **+** | **-** | **-** | 0 | 20MR |
| 23 | CBT83-4 | **+** | **+** | **+** | **+** | 0 | 20MR |
| 24 | CBT83-6 | **+** | **+** | **+** | **+** | 0 | 0 |
| 25 | CBT83-8 | **+** | **+** | **+** | **+** | 0 | 10MR |
| 26 | CBT83-9 | **-** | **-** | **+** | **+** | 3 | 20MR |
| 27 | CBT84-3 | **-** | **-** | **+** | **+** | 0 | 10MR |
| 28 | CBT84-4 | **-** | **-** | **+** | **+** | 0 | 0 |
| 29 | CBT84-5 | **+** | **+** | **+** | **+** | 0 | 0 |
| 30 | CBT84-9 | **-** | **-** | **+** | **+** | 0 | 5MR |
| 31 | CBT84-10 | **-** | **-** | **+** | **+** | 0 | 0 |
| 32 | CBT90-1 | **+** | **+** | **-** | **+** | 0 | 0 |
| 33 | CBT90-3 | **+** | **+** | **-** | **+** | 0 | 10MR |
| 34 | CBT90-4 | **+** | **+** | **-** | **+** | 0 | 20MR |
| 35 | CBT90-5 | **+** | **+** | **-** | **+** | 0 | 0 |
| 36 | CBT90-6 | **+** | **+** | **-** | **+** | 0 | 0 |
| 37 | CBT90-7 | **+** | **+** | **-** | **-** | 0 | 10MR |
| 38 | CBT90-9 | **+** | **+** | **-** | **-** | 0 | 0 |
| 39 | CBT90-10 | **+** | **+** | **-** | **-** | 0 | 0 |
| 40 | CBT90-12 | **+** | **+** | **+** | **+** | 0 | 5MR |
| 41 | CBT90-13 | **+** | **+** | **-** | **-** | 0 | 5MR |
| 42 | CBT90-14 | **+** | **+** | **-** | **-** | 0 | 20MR |
| 43 | CBT90-15 | **+** | **+** | **-** | **-** | 0 | 20MR |
| 44 | CBT90-16 | **+** | **+** | **-** | **-** | 0 | 10MR |
| 45 | CBT91-1 | **+** | **+** | **+** | **+** | 0 | 5MR |
| 46 | CBT91-2 | **+** | **+** | **-** | **-** | 0 | 10MR |
| 47 | CBT91-3 | **+** | **+** | **-** | **-** | 0 | 10MR |
| 48 | CBT91-4 | **-** | **-** | **+** | **+** | 2 | 0 |
| 49 | CBT100-1 | **-** | **-** | **+** | **+** | 0 | 40S |
| 50 | CBT101-1 | **-** | **-** | **+** | **+** | 0 | 0 |
| 51 | CBT101-3 | **-** | **-** | **+** | **+** | 3 | 0 |

^a^ PBW 621 in hexaploid wheat *T. aestivum;* ^b^ ‘+’ and ‘-‘ denote the presence and absence of desirable allele of the gene
